# ﻿Seven new species of the segmented spider genus *Liphistius* (Mesothelae, Liphistiidae) in Thailand and Myanmar

**DOI:** 10.3897/zookeys.1189.115850

**Published:** 2024-01-16

**Authors:** Varat Sivayyapram, Chawakorn Kunsete, Xin Xu, Deborah R. Smith, Prapun Traiyasut, Sureerat Deowanish, Mu Mu Aung, Hirotsugu Ono, Daiqin Li, Natapot Warrit

**Affiliations:** 1 Center of Excellence in Entomology and Department of Biology, Faculty of Science, Chulalongkorn University, Bangkok 10330, Thailand Chulalongkorn University Bangkok Thailand; 2 College of Life Sciences, Hunan Normal University, Changsha, Hunan 410081, China Hunan Normal University Changsha China; 3 Department of Ecology & Evolutionary Biology, Haworth Hall, 1200 Sunnyside Avenue, University of Kansas, Lawrence, KS 66045, USA University of Kansas Lawrence United States of America; 4 Program in Biology, Faculty of Science, Ubon Ratchathani Rajabhat University, Ubon Ratchathani, Thailand Ubon Ratchathani Rajabhat University Ubon Ratchathani Thailand; 5 Forest Research Institute, Forest Department, Ministry of Natural Resources and Environmental Conservation, Yezin, Nay Pyi Taw, Myanmar Ministry of Natural Resources and Environmental Conservation Nay Pyi Taw Myanmar; 6 Department of Zoology, National Museum of Nature and Science, Tsukuba, Ibaraki 305–0005, Japan National Museum of Nature and Science Tsukuba Japan; 7 School of Life Sciences, Hubei University, Wuhan, Hubei 430062, China Hubei University Wuhan, Hubei Province China; 8 Department of Biomedical Science and Environmental Biology, Kaohsiung Medical University, Kaohsiung 80708, Taiwan Kaohsiung Medical University Kaohsiung Taiwan

**Keywords:** Morphology, Southeast Asia, taxonomy, trapdoor spiders

## Abstract

Seven new species of the primitive segmented spider genus *Liphistius* are described and assigned to species groups based on characters of the male palp and vulva plate. The *bristowei* group includes *L.dawei* Sivayyapram & Warrit, **sp. nov.** (♂♀) from southeastern Myanmar, *L.choosaki* Sivayyapram & Warrit, **sp. nov.** (♀) from northwestern Thailand, and *L.lansak* Sivayyapram & Warrit, **sp. nov.** (♀) from western Thailand; the *trang* group (Complex A) contains *L.kaengkhoi* Sivayyapram & Warrit, **sp. nov.** (♂♀), *L.hintung* Sivayyapram & Warrit, **sp. nov.** (♂♀), *L.buyphradi* Sivayyapram & Warrit, **sp. nov.** (♂♀), and *L.champakpheaw* Sivayyapram & Warrit, **sp. nov.** (♂♀) from central Thailand.

## ﻿Introduction

*Liphistius* is the sole genus of the family Liphistiidae (World Spider Catalog 2023), which, with its sister taxon Heptathelidae, comprises the suborder Mesothelae or segmented spiders, the most basal clade of living spiders (Platnick and Gertsch 1976). *Liphistius* retains several characters that are plesiomorphic among spiders, such as presence of abdominal tergites and placement of the spinnerets on the ventral median area of the abdomen (Selden 1996; Xu et al. 2015b; Selden and Ren 2017; Xu et al. 2021). All *Liphistius* species are endemic to Southeast Asia (Indonesia, Laos, Malaysia, Myanmar, Thailand) (World Spider Catalog 2023) except for *Liphistiusnabang* Yu, F. Zhang & J. X. Zhang, 2021 which has been reported from Yunan, in southwestern China (Yu et al. 2021). Currently, 70 *Liphistius* species are recognized and can be classified into seven species groups based on male and female genitalia: the *batuensis* group, *birmanicus* group, *bristowei* group, *linang* group, *malayanus* group, *trang* group, and *tioman* group (Schwendinger 1990, 2017; World Spider Catalog 2023). Here, we describe seven new *Liphistius* species from specimens deposited at the Chulalongkorn University Natural History Museum (CUMZ), Bangkok, Thailand.

## ﻿Materials and methods

A total of 138 *Liphistius* specimens (104 specimens of 29 previously described species and 34 specimens of new species described here) stored in the Museum were examined and dissected for their genitalia under a Zeiss Stemi DV4 stereomicroscope. The specimens were collected between 2017–2021. The male genitalia were examined from the left palp while the vulvae were dissected from the body and cleared by digesting soft tissue using 3M potassium hydroxide. The terminology of the genital characters follows (Schwendinger and Ono 2011; Schwendinger 2017; Schwendinger et al. 2019, 2022). All measurements are reported in millimeters. The spider’s body lengths do not include the chelicerae or anal tubercle. Palp and legs measurements are given in the following format: total length (femur + patella + tibia + metatarsus + tarsus). The specimens were photographed using a Canon EOS 70D DSLR camera attached to a Stackshot Macro Rail (Cognisys Inc., USA). A Canon EF 100mm f/2.8L Macro IS USM lens was attached to the camera for shooting the spider dorsal and lateral habitus. A Laowa 25mm F 2.8 2.5X–5X Macro lens was attached to the camera for shooting the spider genitalia. The photos were recorded in raw file (.CR2) then convert into TIFF file (.tiff) using Canon Digital Professional 4. Multiple digital photos were combined by the focus stacking technique using Zerene Stacker v. 1.04 (Zerene Systems LLC, USA). The stacked photos were developed (combining and removing background) and labelled in Adobe Photoshop and Adobe Illustrator programs. For species identification, the spider morphologies and genitalia were compared with the original descriptions of previously described species. To protect *Liphistius* nesting sites from poaching, the species specific collecting sites and their GPS coordinates are not presented in this article. For more specific information, please contact VS or NW directly. Thai specimens are deposited at CUMZ and the Myanmar specimens will be deposited in the Biodiversity Research Centre of Myanmar which is under construction at the site of the Forest Department, Ministry of Natural Resources and Environmental Conservation at Yezin, Nay Pyi Taw.

## ﻿Comparative material examined

***Liphistiusalbipes* Schwendinger, 1995** – Thailand • 1♂ 2♀; Phra Chaup Khirikhan, Thap Sakae District, along rural road to Chong Lom Water Reservoir; alt. 108 m; 26 November 2017; X. Xu, F. Liu, D. Li, V. Sivayyapram leg.; ARA-2020-051, ARA-2020-052, ARA-2020-053.

***Liphistiusbicoloripes* Ono, 1988** – Thailand • 2♀; Ranong, Suk Samran District, Khlong Na Kha; alt. 52 m; 21 February 2021; V. Sivayyapram leg.; ARA-2021-057, ARA-2021-058.

***Liphistiusbristowei* Platnick & Sedgwick, 1984** – Thailand • 1♀; Chiang Mai, Mueang Chiang Mai District, Suthep; alt. 1110 m; 18 November 2017; X. Xu, F. Liu, D. Li, V. Sivayyapram leg.; ARA-2020-026; • 8♀; Chiang Mai, Mae Chaem District, Tha Pha; alt. 1428 m; 17 June 2019; N. Warrit, V. Sivayyapram, C. Kunsete, W. Nawanetiwong, P. Traiyasut leg.; ARA-2019-028, ARA-2019-032, ARA-2019-039, ARA-2019-040, ARA-2019-044, ARA-2019-046, ARA-2019-048, ARA-2019-050.

***Liphistiuscastaneus* Schwendinger, 1995** – Thailand • 2♀; Ranong, Suk Samran District, Khlong Na Kha; alt. 52 m; 21 February 2021; V. Sivayyapram, leg.; ARA-2021-065, ARA-2021-066.

***Liphistiusdangrek* Schwendinger, 1996** – Thailand • 1♂ 1♀; Ubon Ratchathani, Na Chaluai District, Na Chaluai; alt. 354 m; 11 November 2018; P. Traiyasut leg.; ARA-2020-058, ARA-202-059.

***Liphistiuserawan* Schwendinger, 1996** – Thailand • 2♀; Kanchanaburi, Si Sawat District, Tha Kradan; alt. 272 m; 15 November 2017; X. Xu, F. Liu, D. Li, V. Sivayyapram leg.; ARA-2020-045, ARA-2020-046; • 1♂ 2♀; Kanchanaburi, Si Sawat District, Tha Kradan; alt. 229 m; 20 September 2018; V. Sivayyapram, C. Kunsete, W. Nawanetiwong leg.; ARA-2018-260, ARA-2018-261, ARA-2018-263; • 1♂ 2♀; Kanchanaburi, Sai Yok District, Tha Sao; alt. 380 m; 15 November 2018; V. Sivayyapram, C. Kunsete, W. Nawanetiwong leg.; ARA-2018-314, ARA-2018-315, ARA-2018-319; • 1♂ 2♀; Kanchanaburi, Sai Yok District, Tha Sao; alt. 158 m.; 27 January 2016; N. Warrit, V. Sivayyapram leg.; ARA-2017-118; ARA-2017-125; ARA-2017-127.

***Liphistiusfuscus* Schwendinger, 1995** – Thailand • 2♀; Krabi, Mueang Krabi District, Thab Prik; alt. 307 m; 20 February 2021; V. Sivayyapram leg.; ARA-2020-047, ARA-2020-048.

***Liphistiushatyai* Zhan & Xu, 2022** – Thailand • 2♂ 1♀; Songkhla, Hat Yai District, Kho Hong; alt. 162 m; 13 November 2016; N. Warrit, V. Sivayyapram, N. Chatthanabun, P. Traiyasut leg.; ARA-2017-121, ARA-2017-122, ARA-2017-123.

***Liphistiusindra* Schwendinger, 2017** – Thailand • 5♀; Pattani, Khok Pho District, Sai Khao; alt. 83 m; 17 February 2021; V. Sivayyapram leg.; ARA-2021-001, ARA-2021-004, ARA-2021-006, ARA-2021-007, ARA-2021-010.

***Liphistiusisan* Schwendinger, 1998** – Thailand • 1♀; Sakon Nakhon, Mueang Sakon Nakhon District, Huai Yang; alt. 308 m; 24 May 2018; N. Warrit, V. Sivayyapram, C. Kunsete, W. Nawanetiwong, P. Traiyasut leg.; ARA-2018-194.

***Liphistiusjarujini* Ono, 1988** – Thailand • 1♀; Mueang Tak District, Mae Tho; alt. 881 m; 16 November 2017; X. Xu, F. Liu, D. Li, V. Sivayyapram leg.; ARA-2020-017.

***Liphistiuskeeratikiati* Zhan & Xu, 2022** – Thailand • 3♀; Chumphon, Sawi District, Thung Raya; alt. 48 m; 4 May 2018; N. Warrit, V. Sivayyapram, C. Kunsete, W. Nawanetiwong, P. Traiyasut leg.; ARA-2018-027, ARA-2018-028, ARA-2018-033.

***Liphistiuslahu* Schwendinger, 1998** – Thailand • 1♀; Chiang Mai, Fang District, Ang Kang; alt. 1646 m; 21 November 2017; X. Xu, F. Liu, D. Li, V. Sivayyapram leg.; ARA-2020-028.

***Liphistiusmaewongensis* Sivayyapram** et al., **2017** – Thailand • 1♂ 2♀; Kampang Phet, Klonglan District, Mae Wong National Park, 16.09°N, 99.12°E; alt. 946 m; 4 May 2018; N. Warrit, V. Sivayyapram, N. Chatthanabun leg.; ARA-2017-001, ARA-2017-002, ARA-2017-003.

***Liphistiusmarginatus* Schwendinger, 1990** – Thailand • 1♂ 1♀; Tak, Mueang Tak District, Mae Tho; alt. 868 m; 20 September 2017; N. Chomphuphuang, C. Songsangchote leg.; ARA-2017-124, ARA-2017-125.

***Liphistiusnesioticus* Schwendinger, 1996** – Thailand • 2♀; Trat, Ko Chang District, Ko Chang, along rural road; alt. 84 m; 28 November 2017; X. Xu, F. Liu, D. Li, C. Kunsete leg.; ARA-2020-032, ARA-2020-043.

***Liphistiusniphanae* Ono, 1988** – Thailand • 4♀; Nakhon Si Thammarat, Lansaka District, Khao Kaeo; alt. 112 m; 23 January 2018; N. Warrit, V. Sivayyapram, C. Kunsete, W. Nawanetiwong, P. Traiyasut leg.; ARA-2018-038, ARA-2018-039, ARA-2018-040, ARA-2018-041; • 2♀; Nakhon Si Thammarat, Nopphitam District, Nopphitam; alt. 248 m; 15 February 2021; V. Sivayyapram leg.; ARA-2021-020, ARA-2021-021.

***Liphistiusonoi* Schwendinger, 1996** – Thailand • 3♀; Phitsanulok, Nakhon Thai District, Noen Phoem; alt. 1238 m; June 2017; N. Warrit, V. Sivayyapram, C. Kunsete, W. Nawanetiwong, P. Traiyasut leg.; ARA-2020-055, ARA-2020-056, ARA-2020-057.

***Liphistiusornatus* Ono & Schwendinger, 1990** – Thailand • 7♀; Chanthaburi, Khao Khitchakut District, Pluang; alt. 79 m; 17 March 2018; N. Warrit, V. Sivayyapram, C. Kunsete, N. Chatthanabun, P. Traiyasut leg.; ARA-2018-106, ARA-2018-107, ARA-2018-108, ARA-2018-109, ARA-2018-110, ARA-2018-112, ARA-2018-114; • 1♂; Chanthaburi, Khao Khitchakut District, Pluang; alt. 79 m; 26 October 2021; N. Warrit, C. Kunsete, W. Nawanetiwong leg.; ARA-2021-078.

***Liphistiusphuketensis* Schwendinger, 1998** – Thailand • 3♀; Phuket, Thalang District, Thep Krasatti; alt. 89 m; 23 November 2017; X. Xu, F. Liu, D. Li, V. Sivayyapram leg.; ARA-2020-020, ARA-2020-021, ARA-2020-022.

***Liphistiussayam* Schwendinger, 1998** – Thailand • 2♀; Chon Buri, Si Racha District, Bang Phra; alt. 326 m; 18 March 2018; N. Warrit, V. Sivayyapram, C. Kunsete, N. Chatthanabun, P. Traiyasut leg.; ARA-2018-204, ARA-2018-205.

***Liphistiusschwendingeri* Ono, 1988** – Thailand • 3♀; Ranong, Suk Samran District, Khlong Na Kha; alt. 52 m; 22 January 2018; N. Warrit, V. Sivayyapram, C. Kunsete, W. Nawanetiwong, P. Traiyasut leg.; ARA-2018-027, ARA-2018-028, ARA-2018-033.

***Liphistiustenuis* Schwendinger, 1996** – Thailand • 3♀; Chanthaburi, Laem Sing District, Phliu; alt. 69 m; 6 February 2017; N. Warrit, V. Sivayyapram, P. Traiyasut leg.; ARA-2017-158, ARA-2017-159, ARA-2017-160.

***Liphistiusthaleri* Schwendinger, 2009** – Thailand • 5♀; Trang, Kantang District, Libong Island; alt. 37 m; 18 February 2021; V. Sivayyapram leg.; ARA-2021-022, ARA-2021-023, ARA-2021-024, ARA-2021-025, ARA-2021-028.

***Liphistiustham* Sedgwick & Schwendinger, 1990** – Thailand • 2♀; Saraburi, Kaeng Khoi District, Thap Kwang, Kaeng; alt. 280 m; V. Sivayyapram leg.; ARA-2021-073, ARA-2021-074.

***Liphistiusthoranie* Schwendinger, 1996** – Thailand • 1♂; Nakhon Ratchasima, Mueang Nakhon Nayok, Hin Tung; alt. 1171 m; July 2017; C. Songsangchote leg.; ARA-2020-054; • 1♀; Nakhon Ratchasima, Mueang Nakhon Nayok, Hin Tung; alt. 754 m; 9 October 2016; N. Warrit, V. Sivayyapram, P. Traiyasut leg.; ARA-2020-041.

***Liphistiustrang* Platnick & Sedgwick, 1984** – Thailand • 4♀; Trang, Na Yong District, Chong; alt. 161 m; 23 January 2018; N. Warrit, V. Sivayyapram, C. Kunsete, W. Nawanetiwong, P. Traiyasut leg.; ARA-2018-050, ARA-2018-051, ARA-2018-052, ARA-2018-055.

***Liphistiusyamasakii* Ono, 1988** – Thailand • 5♀; Chiang Mai, Mae Chaem District, Tha Pha; alt. 1428 m; 13 June 2019; N. Warrit, V. Sivayyapram, C. Kunsete, W. Nawanetiwong, P. Traiyasut leg.; ARA-2019-016, ARA-2019-017, ARA-2019-019, ARA-2019-021, ARA-2019-024 • 1♂1♀; Chiang Mai, Mae Chaem District, Tha Pha; alt. 1428 m; 28 October 2020; C. Kunsete, W. Nawanetiwong leg.; ARA-2021-076, ARA-2021-077.

***Liphistiusyangae* Platnick & Sedgwick, 1984** – Thailand • 1♂; Satun, Khuan Don District, Wang Prachan; alt. 117 m; 25 January 2018; N. Warrit, V. Sivayyapram, C. Kunsete, W. Nawanetiwong leg.; ARA-2018-062; • 3♀; Songkhla, Hat Yai District, Hat Yai; alt. 37 m; 30 December 2018; C. Kunsete leg.; ARA-2018-370, ARA-2018-372, ARA-2018-377.

## ﻿Systematics

### ﻿Family Liphistiidae Thorell, 1869

#### ﻿Genus Liphistius

Taxon classificationAnimaliaAraneaeLiphistiidae

Schiødte, 1849

D6EF8D73-12E4-5D2E-9541-11419C077FE8

##### Type species.

*Liphistiusdesultor* Schiødte, 1849.

##### Diagnosis.

*Liphistius* can be distinguished from the heptathelid genera by the male palp possessing a tibial apophysis; the vulva modified into a pore plate or plate-like spermatheca; and the nest structure equipped with signal lines, unique silk lines radiating from the burrow entrance (Platnick and Sedgwick 1984; Xu et al. 2015a).

##### Distribution.

China (Yunnan Province), Indonesia (Sumatra), Laos, Peninsular Malaysia, Myanmar, and Thailand.

#### ﻿ Liphistius dawei

Taxon classificationAnimaliaAraneaeLiphistiidae

Sivayyapram & Warrit sp. nov.

CD688CF0-5F6A-539D-A44F-37C5CE6D9EAF

https://zoobank.org/9F369E2C-F3DB-4F63-ADE1-E4FD4C7F063B

Figs 1 , 2 , 3

##### Type material.

***Holotype***: Myanmar • 1♂; Dawei, Pa Kar Ri; alt. 20 m; 4 May 2018; N. Warrit, V. Sivayyapram, C. Kunsete, N. Chatthanabun, P. Traiyasut leg.; ARA-2018-143. ***Allotype***: Myanmar • 1♀; same data as for the holotype; ARA-2018-138. ***Paratypes***: Myanmar • 1♂ 5♀; same data as for the holotype; ARA-2018-136, ARA-2018-137, ARA-2018-139, ARA-2018-140, ARA-2018-144, ARA-2018-147.

**Figure 1.**
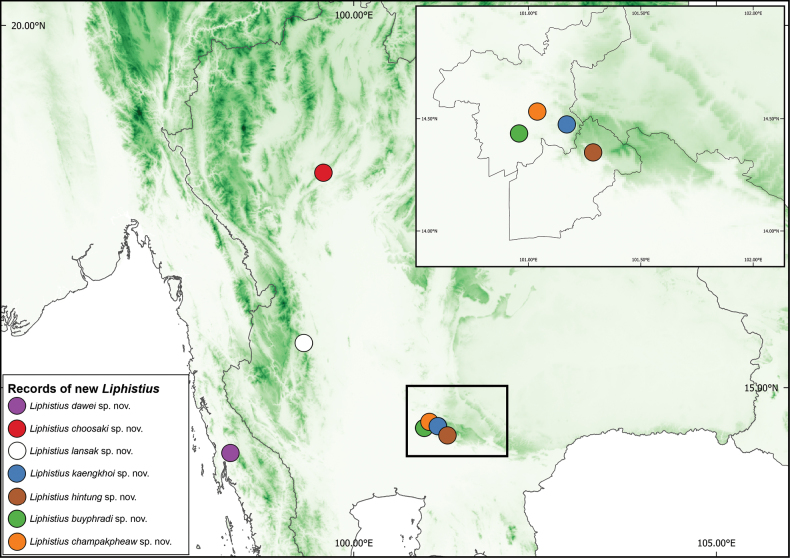
Map showing the localities of the new *Liphistius* species described.

##### Other material.

Myanmar • 3 juveniles; same data as for the holotype; ARA-2018-146, ARA-2018-148, ARA-2018-149.

##### Diagnosis.

*Liphistiusdawei* sp. nov. is similar to *L.inthanon* Zhan & Xu, 2022 and *L.yamasakii* Ono, 1988 in large body size with uniformly dark color. The male of *L.dawei* sp. nov. can be distinguished from those of *L.inthanon* and *L.yamasakii* by the palp: subtegulum with moderate apophysis, not enlarged at the tip; tegulum with finer dentate edge of proximal margin and more pronounced marginal apophysis (Fig. 3A–D; Ono 1988: fig. 8; Schwendinger 1990: fig. 18; Zhan et al. 2022: fig. 4A–G). The female of *L.dawei* sp. nov. can be distinguished from those of *L.inthanon* and *L.yamasakii* by the vulva: posterior stalk axe-blade shaped, constricted at the base; pore plate with less projecting posterior corners of the lateral lips (Fig. 3E, F; Ono 1988: figs 6, 7; Schwendinger 1990: fig. 19; Zhan et al. 2022: fig. 4H–M).

##### Description.

**Figure 2.**
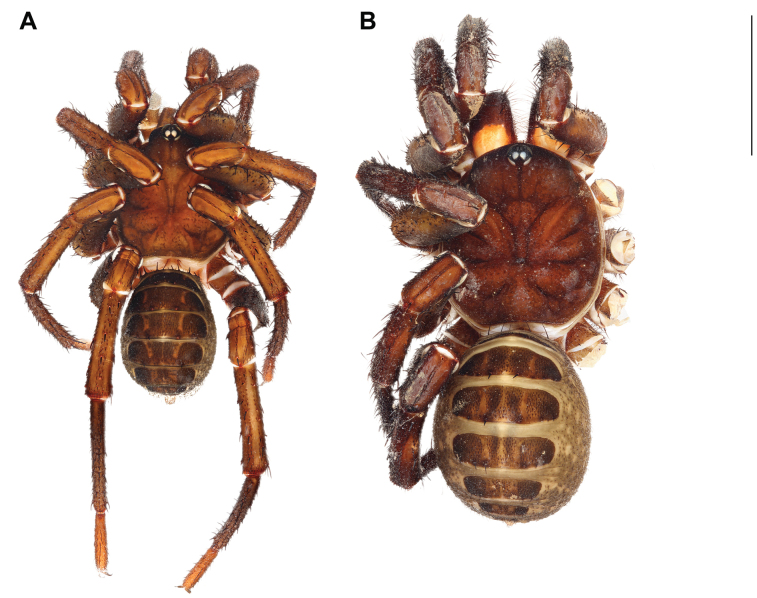
*Liphistiusdawei* sp. nov. dorsal habitus **A** male, ARA-2018-143 (holotype) **B** female, ARA-2018-138 (allotype). Scale bar: 10 mm.

**Male** (Holotype: ARA-2018-143; Fig. 2A). ***Coloration (in alcohol)***: carapace uniformly brown, with black stripe along the margins, bearing short black setae on cephalic region and coxal elevations; abdominal tergite brown, bearing short black setae, paler on the glabrous area; membranous part of the opisthosoma cream color with a smear of black pigment; chelicerae pale brown; palp and legs pale brown, without distinct annulations.

**Figure 3.**
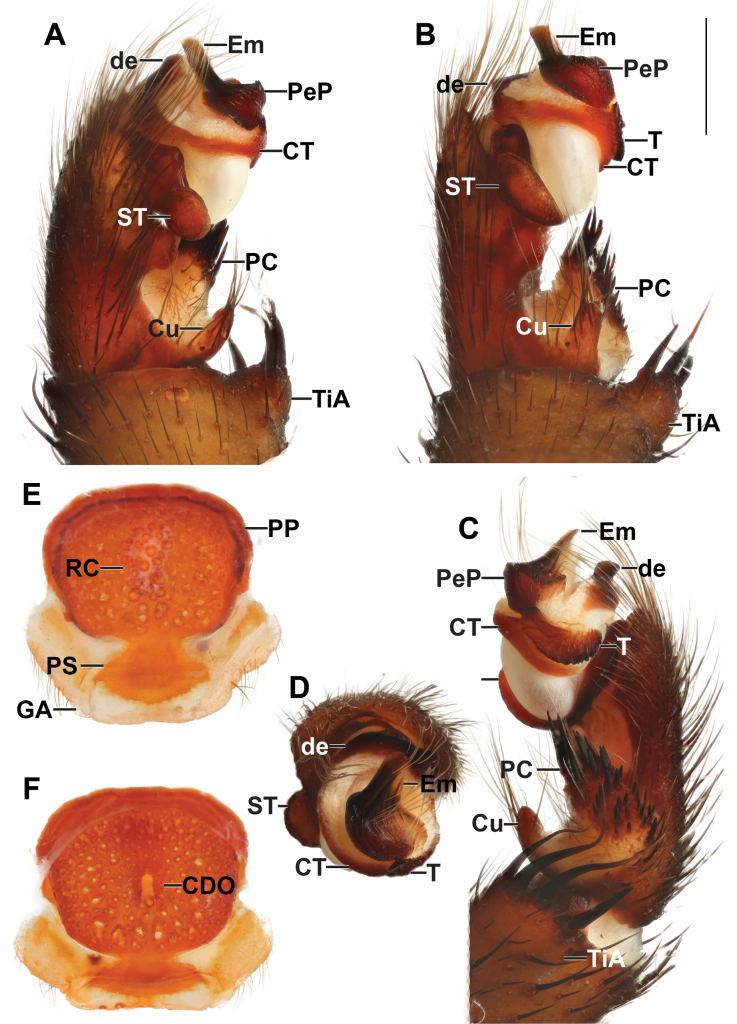
*Liphistiusdawei* sp. nov. male palp and vulva plate **A–D** ARA-2018-143 (holotype) palp **A** prolateral view **B** ventral view **C** retrolateral view **D** distal view **E, F** ARA-2018-138 (allotype) vulva plate **E** ventral view **F** dorsal view. Abbreviations: CDO = central dorsal opening; CT = contrategulum; Cu = cumulus; de = distal edge of the contrategulum; Em = embolus; GA = genital atrium; mm = millimeter; PC = paracymbium; PeP = paraembolic plate; PP = poreplate; PS = posterior stalk; RC = receptacular cluster; ST = subtegulum; T = tegulum; TiA = tibial apophysis. Scale bar: 1 mm.

***Palp*** (Fig. 3A–D): tibial apophysis short, truncate, carrying four black tapering megaspines; cumulus distinctly elevated, bearing long black bristles; paracymbium long, narrow, dark patch with spicules partially isolated by a pale band; subtegulum with moderated apophysis, not enlarged at the tip; contrategulum without apophysis, distal edge of contrategulum arched, leading to conical apex; tegulum kidney-shaped, wider than long, proximal margin convex with finely dentate edge, distal margin slightly concave with round and pronounced apophysis; pigmental bridge between contrategulum and tegulum indistinct; paraembolic plate indistinct, not projecting into a scale-like plate, sclerotized part of the embolus with two longitudinal ridges reaching to the tip.

***Measurements***: Total length 20.15; carapace 9.75 long, 9.10 wide; opisthosoma 9.36 long, 7.28 wide; ocular tubercle 1.30 long, 1.56 wide; palpal coxa 3.12 long, 1.82 wide; labium 1.04 long, 1.82 wide; sternum 4.55 long, 2.73 wide (1.43 on ventral surface); palp 16.90 long (5.59 + 3.38 + 5.33 + – + 2.60); leg I 28.21 long (8.58 + 4.16 + 5.85 + 7.02 + 2.60); leg II 30.42 long (8.97 + 4.03 + 6.50 + 7.93 + 2.93); leg III 32.04 long (8.58 + 4.16 + 6.50 + 7.93 + 2.93); leg IV 39.91 long (10.7 + 4.29 + 8.19 + 12.48 + 4.68).

**Female** (Allotype: ARA-2018-138; Fig. 2B). ***Coloration (in alcohol)***: carapace uniformly brown, bearing short black setae on the cephalic region and coxal elevations; abdominal tergites brown, darker in the area with short black setae; membranous part of the opisthosoma cream colored with thin layer of smear black marking; chelicerae bicolor, orange on proximal part and brown on distal part; palp and legs brown, without distinct annulations.

***Vulva*** (Fig. 3E, F): vulva plate hexagonal, genital atrium with folded lateral margins, carrying lateral hairs; posterior stalk axe-blade shaped, constricted at the based, posterior margin convex; pore plate rectangular and wider than long, lateral margin thickened and projecting into a lips, more distinct on anterior portion, posterior corner slightly projecting, anterior margin thickened and projecting into a lip, slightly arched; receptacular cluster racemose, longer than wide; central dorsal opening wide longer than wide.

***Measurements***: Total length 27.43; carapace 12.87 long, 12.09 wide; opisthosoma 14.04 long, 11.44 wide; ocular tubercle 1.69 long, 1.95 wide; palpal coxa 4.42 long, 2.47 wide; labium 1.82 long, 3.12 wide; sternum 6.24 long, 3.51 wide (2.08 on ventral surface); palp 22.23 long (8.06 + 4.16 + 5.07 + – + 4.94); leg I 28.47 long (9.75 + 4.94 + 5.98 + 5.46 + 2.34); leg II 29.51 long (9.62 + 5.07 + 5.98 + 5.72 + 3.12); leg III 30.42 long (8.97 + 5.33 + 5.85 + 7.28 + 2.99); leg IV 41.34 long (11.44 + 5.46 + 7.80 + 12.09 + 4.55).

##### Etymology.

The specific epithet *dawei* refers to the type locality of the new species in Dawei State, Myanmar.

##### Distribution.

Known only from the type locality.

##### Comment.

The new species was mentioned as *Liphistius* sp. DW in Sivayyapram et al. (2023).

#### ﻿ Liphistius choosaki

Taxon classificationAnimaliaAraneaeLiphistiidae

Sivayyapram & Warrit sp. nov.

3E9526E8-9913-578A-A058-70431C379398

https://zoobank.org/B14F4BAB-5156-479D-93E0-5CDE9F8C2A50

Figs 1 , 4

##### Type material.

***Holotype***: Thailand • 1♀; Phrae, Wang Chin District, Mae Koeng; alt. 265 m; 5 October 2019; N. Warrit, V. Sivayyapram, C. Kunsete, N. Chatthanabun, P. Traiyasut leg.; ARA-2019-057. ***Paratype***: Thailand • 1♀; same data as for the holotype; ARA-2019-056.

##### Other materials.

Thailand • 3 juveniles; same data as for the holotype; ARA-2019-059, ARA-2019-061, ARA-2019-062.

##### Diagnosis.

*Liphistiuschoosaki* sp. nov. is similar to *L.dawei* sp. nov., *L.inthanon*, and *L.yamasakii* in its uniformly dark coloration. The female of *L.choosaki* sp. nov. can be distinguished from those of *L.dawei*, *L.inthanon*, and *L.yamasakii* by the characters of vulva: pore plate distinctly wider than long with almost straight anterior margin; and by its larger body size.

##### Description.

**Male.** Unknown.

**Figure 4.**
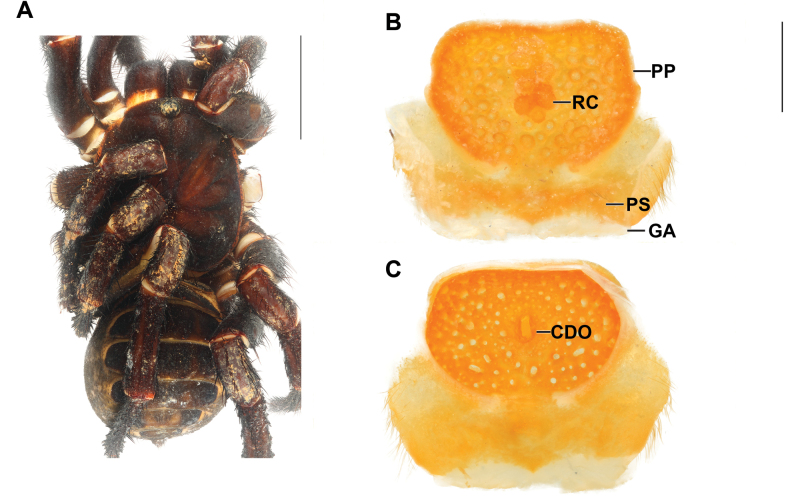
*Liphistiuschoosaki* sp. nov. female, ARA-2019-057 (holotype) **A** dorsal view **B, C** vulva plate **B** ventral view **C** dorsal view. Abbreviations: CDO = central dorsal opening; GA = genital atrium; mm = millimeter; PP = poreplate; PS = posterior stalk; RC = receptacular cluster. Scale bars: 10 mm (**A**); 1 mm (**B, C**).

**Female** (Holotype: ARA-2019-057; Fig. 4A). ***Coloration (in alcohol)***: carapace uniformly brown; abdominal tergites dark brown; membranous part of the opisthosoma cream color with thin mottled black marking; chelicerae bicolor, orange on proximal part and dark brown on distal part; palp and legs brown, without distinct annulations.

***Vulva*** (Fig. 4B, C): vulva plate hexagonal, genital atrium with folded lateral margins, carrying lateral hairs; posterior stalk very wide, M-shaped posterior margin; pore plate rectangular, wider than long, lateral margin convex, anterior margin almost straight, all margins thickened and projected into a lip; receptacular cluster racemose, longer than wide; central dorsal opening wide longer than wide.

***Measurements***: Total length 36.40; carapace 18.33 long, 16.38 wide; opisthosoma 17.94 long, 15.34 wide; ocular tubercle 1.82 long, 1.95 wide; palpal coxa 5.46 long, 3.25 wide; labium 2.08 long, 4.29 wide; sternum 9.75 long, 3.90 wide (1.95 on ventral surface); palp 29.12 long (10.66 + 5.59 + 7.02 + – + 5.85); leg I 34.71 long (12.48 + 6.50 + 7.15 + 6.24 + 2.34); leg II 36.40 long (11.96 + 5.85 + 7.41 +7.54 +3.64); leg III 40.82 long (12.74 + 6.89 + 7.67 + 9.49 + 4.03); leg IV 53.69 long (15.21 + 7.41 + 10.79 + 14.95 + 5.33).

##### Etymology.

The specific epithet *choosaki* honors the late Mr. Choosak Pungrusmee, father to Mr. Sarawut Pungrusmee and dedicated philanthropist to the study of biodiversity in our research laboratory.

##### Distribution.

Known only from the type locality.

##### Comment.

This new species name was mentioned as *Liphistius* sp. WKS in Sivayyapram et al. (2023).

#### ﻿ Liphistius lansak

Taxon classificationAnimaliaAraneaeLiphistiidae

Sivayyapram & Warrit sp. nov.

2238C539-3A36-5121-9CEB-CA60115E69AC

http://zoobank.org/49787452-9ACB-4BB4-AD78-006CE5A02D25

Figs 1 , 5

##### Type material.

***Holotype***: Thailand • 1♀; Uthai Thani, Lan Sak District, Rabam; alt. 200 m; 29 October 2020; V. Sivayyapram leg.; ARA-2021-067. ***Paratype***: Thailand • 1♀, same data as for the holotype; ARA-2021-068.

##### Diagnosis.

*Liphistiuslansak* sp. nov. is a small *Liphistius* species recognized by the unique vulva: pore plate with receptacular cluster flanked by a pair of large vesicles.

##### Description.

**Male.** Unknown.

**Figure 5.**
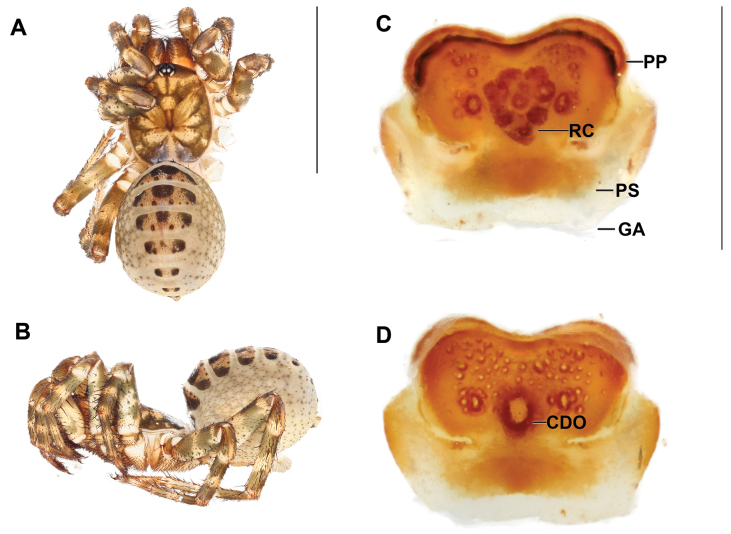
*Liphistiuslansak* sp. nov. **A, B** female, ARA-2021-068 (holotype) **A** dorsal view **B** lateral view **C, D** vulva plate **C** ventral view **D** dorsal view. Abbreviations: CDO = central dorsal opening; GA = genital atrium; mm = millimeter; PP = poreplate; PS = posterior stalk; RC = receptacular cluster. Scale bars: 10 mm (**A, B**); 1 mm (**C, D**).

**Female** (Holotype: ARA-2021-067; Fig. 5A, B). ***Coloration (in alcohol)***: carapace pale brown with black marking on the cephalic region and the margin of the thoracic region; abdominal tergites pale brown, with black marking on the anterior and lateral margins of each plate; membranous part of the opisthosoma cream colored with thin mottled black marking; chelicerae dark brown, with black marking, except on the proximal part; palp and legs pale brown, with black annulations on the proximal and distal part of each joint (Fig. 5B).

***Vulva*** (Fig. 5C, D): vulva plate hexagonal, wider than long, genital atrium without lateral hair; posterior stalk short but wide, posterior margin W-shaped; pore plate rectangular, wider than long, lateral margin not projected into a lip, anterior margin invaginated, thickened and projected into a lip; receptacular cluster racemose, as long as wide, flanked by a pair of large vesicles; central dorsal opening round and wide.

***Measurements***: Total length 14.17; carapace 5.98 long, 5.46 wide; opisthosoma 8.19 long, 6.89 wide; ocular tubercle 0.91 long, 1.04 wide; palpal coxa 2.21 long, 1.17 wide; labium 0.78 long, 1.82 wide; sternum 3.12 long, 1.95 wide (1.30 on ventral surface); palp 10.79 long (3.77 + 1.95 + 2.47 + – + 2.60); leg I 14.04 long (4.55 + 2.08 + 2.86 + 2.99 + 1.56); leg II 15.08 long (4.81 + 2.21 + 2.99 + 3.38 + 1.56); leg III 17.16 long (4.68 + 2.47 + 3.25 + 4.03 + 2.73); leg IV 20.94 long (5.33 + 2.47 + 4.03 + 5.98 + 3.13).

##### Etymology.

The specific epithet *lansak* refers to Lan Sak District, the type locality of the new species in Uthai Thani, Thailand.

##### Distribution.

Known only from the type locality.

##### Comment.

This new species name was mentioned as *Liphistius* sp. HKK in Sivayyapram et al. (2023).

#### ﻿ Liphistius kaengkhoi

Taxon classificationAnimaliaAraneaeLiphistiidae

Sivayyapram & Warrit sp. nov.

74A1866D-A8BC-5195-A754-12C5A658644C

https://zoobank.org/BA62345B-B964-4845-B248-147400A0FAE6

Figs 1 , 6 , 7 , 14

##### Type material.

***Holotype***: Thailand • 1♂; Saraburi, Kaeng Khoi District, Cha Om; alt. 127 m; 14 October 2018; N. Warrit, V. Sivayyapram, C. Kunsete, N. Chatthanabun, P. Traiyasut leg.; ARA-2018-284. ***Allotype***: Thailand • 1♀, same data as for the holotype; ARA-2018-286. ***Paratypes***: Thailand • 2♂ 4♀; same data as for the holotype; ARA-2018-281, ARA-2018-282, ARA-2018-283, ARA-2018-285, ARA-2018-289, ARA-2018-291.

##### Diagnosis.

*Liphistiuskaengkhoi* sp. nov. is similar to *L.buyphradi* sp. nov., *L.champakpheaw* sp. nov., *L.hintung* sp. nov., and *L.suwat* Schwendinger, 1996 in general appearance. The male of *L.kaengkhoi* sp. nov. can be distinguished from that of *L.suwat* by the paracymbium not bent outward; and the contrategulum without short blunt cone (Fig. 7; Schwendinger 1996: figs 43, 43A); from those of *L.buyphradi* sp. nov. and *L.champakpheaw* sp. nov. by the male palp with swollen paracymbium (flat in *L.buyphradi* sp. nov., *L.champakpheaw* sp. nov.; Figs 11, 13); *L.kaengkhoi* sp. nov. is very similar to *L.hintung* sp. nov. but can be distinguished by the shorter and finer dentate edge on the proximal margin of the tegulum and shorter paraembolic plate (Figs 7A–D, 9A–D). The female *L.kaengkhoi* sp. nov. is difficult to distinguish from those of the *L.buyphradi* sp. nov., *L.champakpheaw* sp. nov., *L.hintung* sp. nov., and *L.suwat* Schwendinger, 1996. Molecular phylogeny and species delimitation using *COI* and multi-locus data support monophyly and species status of all new species described here (Sivayyapram et al. 2023).

##### Description.

**Figure 6.**
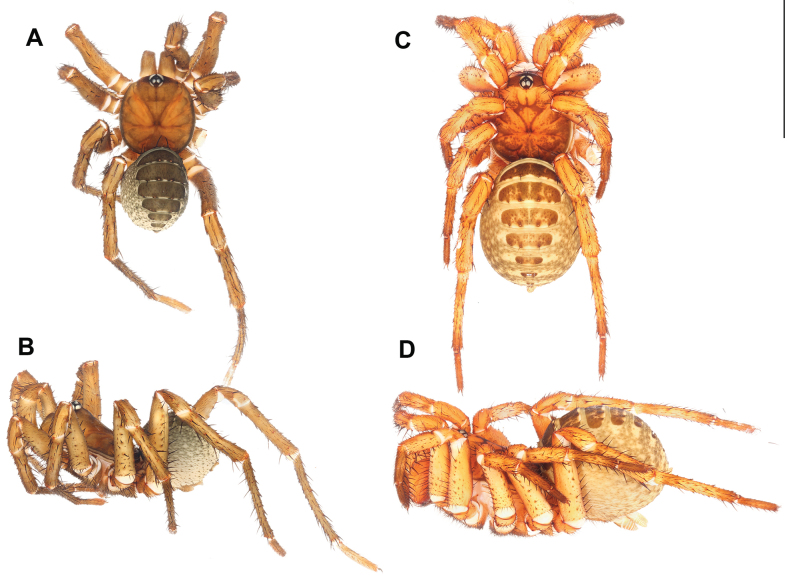
*Liphistiuskaengkhoi* sp. nov. **A, B** male ARA-2018-284 (holotype) **C, D** female, ARA-2018-286 (allotype) **A, C** dorsal view **B, D** lateral view. Scale bar: 10 mm.

**Male** (Holotype: ARA-2018-284; Fig. 6A, B). ***Coloration (in alcohol)***: carapace brown with indistinct black mottling on cephalic region and coxal elevations; abdominal tergites black; membranous part of the opisthosoma cream colored with black mottling on antero-dorsal portion; chelicerae brown, paler on proximal portion; palp and legs brown without distinct annulation.

**Figure 7.**
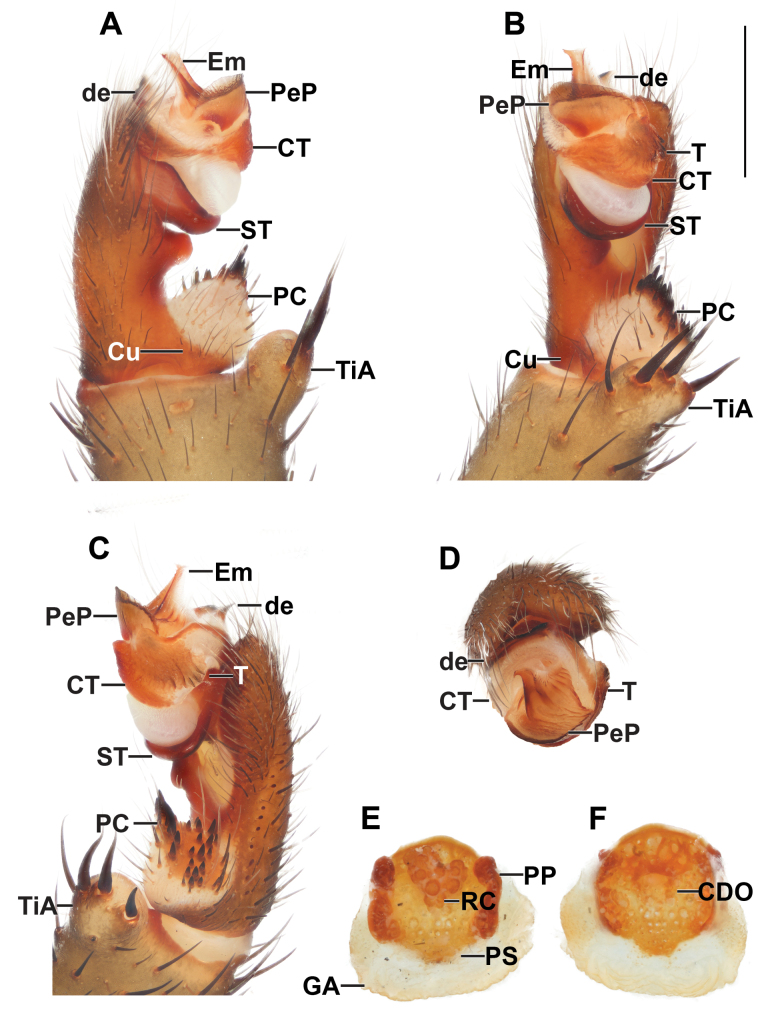
*Liphistiuskaengkhoi* sp. nov. male palp and vulva plate **A–D** ARA-2018-284 (holotype) palp **A** prolateral view **B** ventral view **C** retrolateral view **D** distal view **E, F** ARA-2018-286 (allotype) vulva plate **E** ventral view **F** dorsal view. Abbreviations: CDO = central dorsal opening; CT = contrategulum; Cu = cumulus; de = distal edge of the contrategulum; Em = embolus; GA = genital atrium; mm = millimeter; PC = paracymbium; PeP = paraembolic plate; PP = poreplate; PS = posterior stalk; RC = receptacular cluster; ST = subtegulum; T = tegulum; TiA = tibial apophysis. Scale bar: 1 mm.

***Palp*** (Fig. 7A–D): tibial apophysis large, carrying one long slender and three tapering megaspines; paracymbium short, almost round, dark patch with spicules isolated by a pale band; cumulus plain, bearing thin black bristles; subtegulum without apophysis; contrategulum without apophysis, distal edge of contrategulum long, slightly invaginate leading to the conical apex; tegulum large, axe-blade shaped, indistinctly separated from the contrategulum by corrugated surface, proximal margin with a short moderate dentate edge, distal margin almost straight, with round apophysis; pigmental bridge between tegulum and contrategulum sigmoid in shape; paraembolic plate projecting to a scale-liked plate, basally wide, leading into short triangular distal margin; embolus proper: sclerotized part with two longitudinal ridges reaching to the truncated apex.

***Measurements***: Total length 11.44; carapace 5.85 long, 5.72 wide; opisthosoma 5.82 long, 5.07 wide; ocular tubercle 0.97 long, 1.17 wide; palpal coxa 2.21 long, 1.30 wide; labium 0.65 long, 1.3 wide; sternum 3.99 long, 1.95 wide (0.91 on ventral surface); palp 12.09 long (3.90 + 2.21 + 3.90 + – + 2.08); leg I 18.72 long (5.72 + 2.73 + 3.77 + 4.42 + 2.08); leg II 19.50 long (5.72 + 2.60 + 3.90 + 4.81 + 2.47); leg III 20.80 long (5.46 + 2.60 + 4.29 + 5.85 + 2.60); leg IV 26.25 long (6.89 + 2.99 + 5.20 + 7.67 + 3.51).

**Female** (Allotype: ARA-2018-286; Fig. 6C, D). ***Coloration (in alcohol)***: carapace orange with thick black band on the anterior margin and black mottled marking in the posterior portion of the cephalic region and the thoracic region; abdominal tergites cream colored with large mottled black markings; membranous part of the opisthosoma cream with black mottled marking; chelicerae orange; palp and legs: femur to tibia orange, tarsi and metatarsi of legs I–III black, metatarsi of leg IV orange with black annulation on the proximal and distal area.

***Vulva*** (Fig. 7E, F): vulva plate almost round, genital atrium without lateral hair; posterior stalk short, V-shaped; pore plate rectangular, lateral margins thickened and project into lips, anterior margin convex, less thicken, not project into a lip; receptacular clusters racemose, grape-like in shape; central dorsal opening wide.

***Measurements***: Total length 16.2; carapace 6.89 long, 6.50 wide; opisthosoma 9.49 long, 9.67 wide; ocular tubercle 1.04 long, 1.04 wide; palpal coxa 2.60 long, 1.56 wide; labium 0.78 long, 1.95 wide; sternum 3.64 long, 2.21 wide (1.30 on ventral surface); palp 12.87 long (4.68 + 2.47 + 2.86 + – + 2.86); leg I 15.47 long (5.20+ 2.86 + 2.99 + 2.86 + 1.56); leg II 15.99 long (5.20 + 2.73 + 2.99 + 3.25 + 1.82); leg III 16.64 long (5.07 + 2.60 + 3.12 + 3.77 + 2.08); leg IV 23.81 long (7.02 + 3.38 + 4.18 + 6.24 + 2.99).

##### Etymology.

The specific epithet *kaengkhoi* refers to Kaeng Khoi District, the type locality of the new species in Saraburi, Thailand.

##### Distribution.

Known only from the type locality.

##### Comment.

This new species name was mentioned as *Liphistius* sp. CK in Sivayyapram et al. (2023).

#### ﻿ Liphistius hintung

Taxon classificationAnimaliaAraneaeLiphistiidae

Sivayyapram & Warrit sp. nov.

EA7D1033-369F-52DB-BB37-51E083F2535B

https://zoobank.org/05DD8E71-5B21-4756-ACB9-9A1C3B5E62F3

Figs 1 , 8 , 9 , 14

##### Type material.

***Holotype***: Thailand • 1♂; Nakhon Nayok, Mueang Nakhon Nayok District, Hin Tung; alt. 90 m; 27 November 2018; D. Li, L. Yu V. Sivayyapram leg.; ARA-2018-299. ***Allotype***: Thailand • 1♀; same data as for the holotype; ARA-2018-296. ***Paratype***: Thailand • 1 juvenile; same data as for the holotype; ARA-2018-297.

##### Diagnosis.

*Liphistiushintung* sp. nov. is similar to *L.kaengkhoi* sp. nov., *L.buyphradi* sp. nov., *L.champakpheaw* sp. nov., and *L.suwat* in its general appearance. *Liphistiushintung* sp. nov. is closely similar to *L.kaengkhoi* sp. nov. but can be distinguished by the male palp: tegulum with coarser proximal dental edge and longer paraembolic plate (Figs 7A–D, 9A–D). The female *L.hintung* sp. nov. is difficult to distinguish from those of the *L.kaengkhoi* sp. nov., *L.buyphradi* sp. nov., *L.champakpheaw* sp. nov., and *L.suwat* Schwendinger, 1996. Molecular phylogeny and species delimitation using *COI* and multi-locus data support monophyly and species status of all new species described here (Sivayyapram et al. 2023).

##### Description.

**Figure 8.**
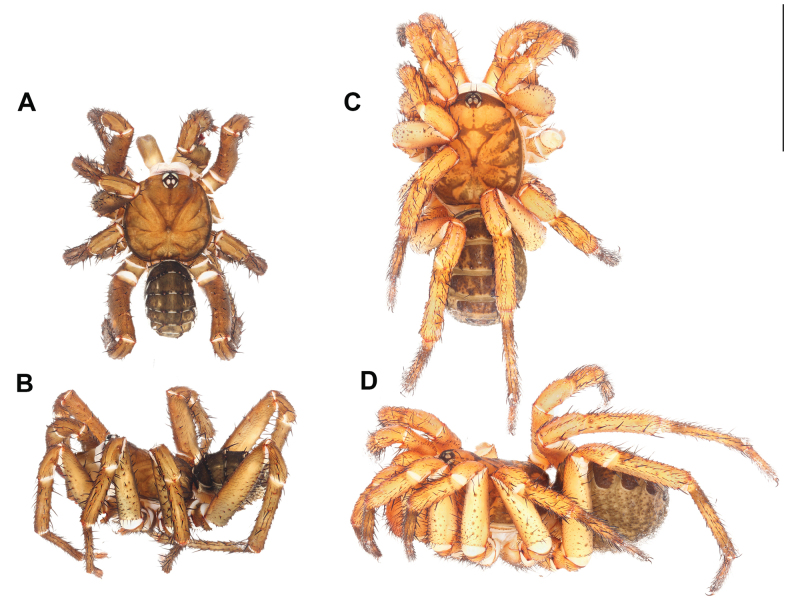
*Liphistiushintung* sp. nov. **A, B** male ARA-2018-299 (holotype) **C, D** female, ARA-2018-296 (allotype) **A, C** dorsal view **B, D** lateral view. Scale bar: 10 mm.

**Male** (Holotype: ARA-2018-299; Fig. 8A, B). ***Coloration (in alcohol)***: carapace pale brown, with black stripe along the lateral and posterior margins; abdominal tergites almost black, paler on the posterior ones; membranous part of the opisthosoma cream in color with black mottled spots; chelicerae olive green, paler at the proximal part; palp and legs uniformly brown.

**Figure 9.**
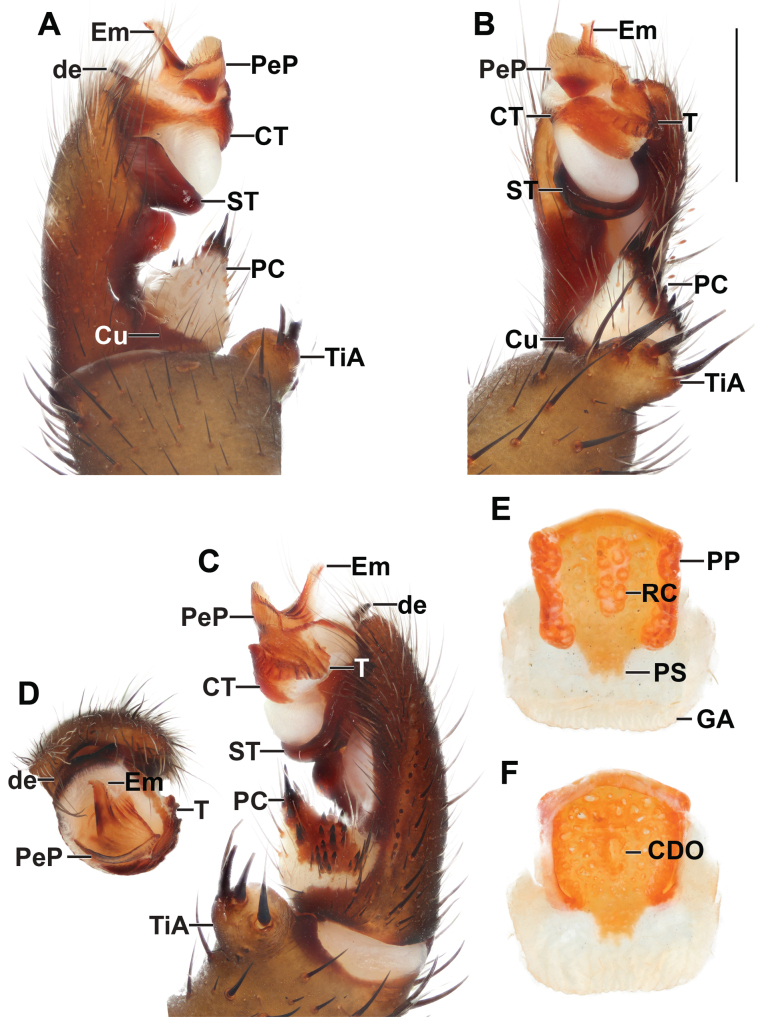
*Liphistiushintung* sp. nov. male palp and vulva plate **A–D** ARA-2018-299 (holotype) palp **A** prolateral view **B** ventral view **C** retrolateral view **D** distal view **E, F** ARA-2018-296 (allotype) vulva plate **E** ventral view **F** dorsal view. Abbreviations: CDO = central dorsal opening; CT = contrategulum; Cu = cumulus; de = distal edge of the contrategulum; Em = embolus; GA = genital atrium; mm = millimeter; PC = paracymbium; PeP = paraembolic plate; PP = poreplate; PS = posterior stalk; RC = receptacular cluster; ST = subtegulum; T = tegulum; TiA = tibial apophysis. Scale bar: 1 mm.

***Palp*** (Fig. 9A–D): tibial apophysis pronounce, carrying one long slender and three tapering megaspines; paracymbium conical, dark patch with spicules isolated by a pale band; cumulus plain, bearing some bristles; subtegulum without apophysis; contrategulum without apophysis, distal edge of contrategulum long and thick, slightly concave leading to the blunt apex; tegulum large, indistinctly separated from the contrategulum, axe-blade shaped, proximal margin with moderate long, coarsely dentate edge, distal margin oblique with large apophysis; pigmental bridge between the tegulum and contrategulum distinct; paraembolic plate projected into scale-like plate, basally wide with long and pointed distal edge; embolus proper: sclerotized part with two longitudinal ridges running to the truncate apex.

***Measurements***: Total length 12.09; carapace 6.76 long, 6.37 wide; opisthosoma 5.33 long, 3.64 wide; ocular tubercle 1.17 long, 1.17 wide; palpal coxa 2.21 long, 1.56 wide; labium 0.78 long, 1.82 wide; sternum 3.64 long, 1.95 wide (1.04 on ventral surface); palp 12.35 long (3.90 + 2.34 + 4.03 + – + 2.08); leg I 19.37 long (5.85 + 2.99 + 3.90 + 4.42 + 2.21); leg II 19.89 long (5.72 + 2.73 + 4.03 + 5.20 + 2.21); leg III 22.88 long (6.11 +3.12 + 4.55 + 6.37 + 2.73); leg IV 27.81 long (7.14 + 3.25 + 5.72 + 8.45 + 3.25).

**Female** (Allotype: ARA-2018-296; Fig. 8C, D). ***Coloration (in alcohol)***: carapace orange, with black marking behind the ocular tubercle running to the fovea and coxal elevations and black stripe along the carapace margins, thicker on the anterior margin; abdominal tergites pale brown with large black marking; membranous part of the opisthosoma cream colored with mottled black spots; palp and legs orange with black annulations on the metatarsus and tarsus.

***Vulva*** (Fig. 9E, F): vulva plate hexagonal; genital atrium with a few hairs; posterior stalk trapezoidal, wider anteriorly; pore plate almost square, lateral margins thickened and projected into a lip, bearing indistinct anterolateral lobes; anterior margin convex, less thicken and not project into a lip; receptacular clusters racemose, longer than wide; central dorsal opening wide, longer than wide.

***Measurements***: Total length 16.90; carapace 8.06 long, 7.02 wide; opisthosoma 8.32 long, 6.63 wide; ocular tubercle 1.17 long, 1.17 wide; palpal coxa 2.60 long, 1.43 wide; labium 1.04 long, 2.08 wide; sternum 3.77 long, 2.60 wide (1.56 on ventral surface); palp 13.91 long (4.94 + 2.47 + 3.38 + – + 3.12); leg I 16.90 long (5.59 + 3.12 + 3.38 + 3.12 + 1.69); leg II 17.81 long (5.72 + 2.99 + 3.77 + 3.51 + 1.82); leg III 18.72 long (5.59 + 3.12 + 3.77 + 4.16 + 2.08); leg IV 26.26 long (7.54 + 3.51 + 5.20 + 6.63 + 3.38).

##### Etymology.

The specific epithet *hintung* refers to Hintung District, the type locality of the new species in Nakhon Nayok, Thailand.

##### Distribution.

Known only from the type locality.

##### Comment.

This new species name was mentioned as *Liphistius* sp. WTK in Sivayyapram et al. (2023)

#### ﻿ Liphistius buyphradi

Taxon classificationAnimaliaAraneaeLiphistiidae

Sivayyapram & Warrit sp. nov.

99B3C343-7DA2-5C69-AA3D-F5C5ED1B6E8D

https://zoobank.org/FFFB3423-C421-48AE-858F-6D1B8D169082

**Figure 10.**
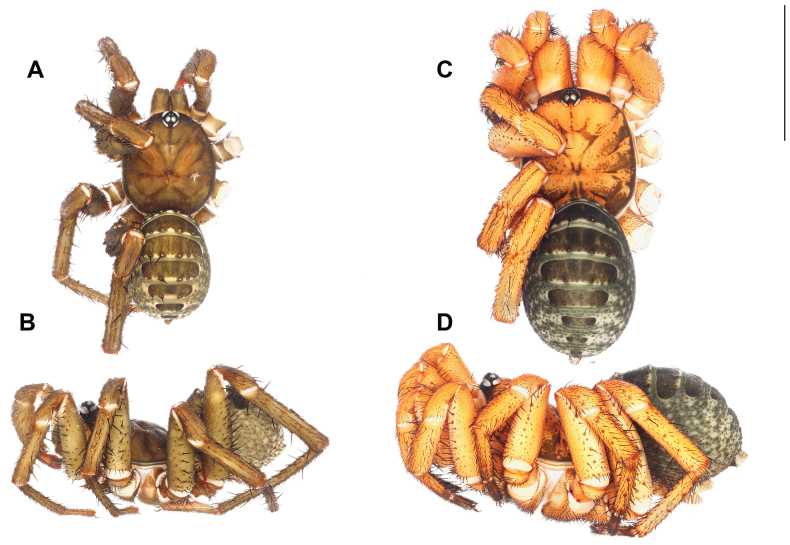
*Liphistiusbuyphradi* sp. nov. **A, B** male ARA-2017-139 (holotype) **C, D** female, ARA-2017-140 (allotype) **A, C** dorsal view **B, D** lateral view. Scale bar: 10 mm.

**Figure 11.**
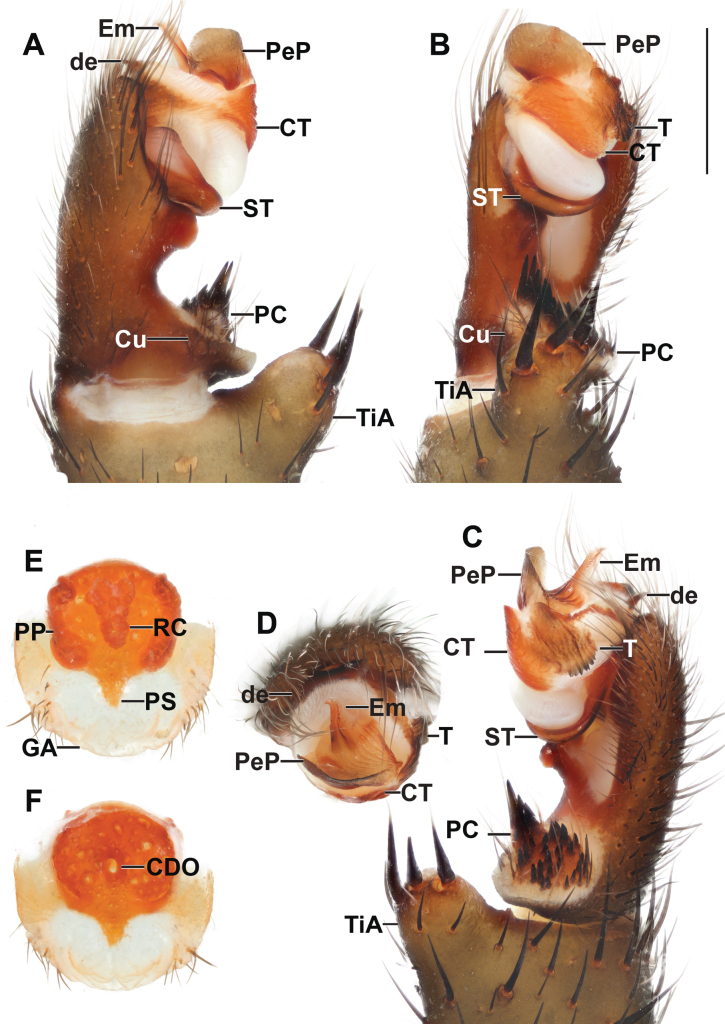
*Liphistiusbuyphradi* sp. nov. male palp and vulva plate **A–D** ARA-2017-139 (holotype) palp **A** prolateral view **B** ventral view **C** retrolateral view **D** distal view **E, F** ARA-2017-140 (allotype) vulva plate **E** ventral view **F** dorsal view. Abbreviations: CDO = central dorsal opening; CT = contrategulum; Cu = cumulus; de = distal edge of the contrategulum; Em = embolus; GA = genital atrium; mm = millimeter; PC = paracymbium; PeP = paraembolic plate; PP = poreplate; PS = posterior stalk; RC = receptacular cluster; ST = subtegulum; T = tegulum; TiA = tibial apophysis. Scale bar: 1 mm.

Figs 1 , 10 , 11 , 14

##### Type material.

***Holotype***: Thailand • 1♂; Saraburi, Mueang Saraburi District, Nong Pla Lai; alt. 90 m; 17 August 2017; N. Warrit, V. Sivayyapram, C. Kunsete, N. Chatthanabun, P. Traiyasut leg.; ARA-2017-139. ***Allotype***: Thailand • 1♀; same data as for the holotype; ARA-2017-140. ***Paratype***: Thailand • 1♀; same data as for the holotype; ARA-2017-138.

##### Diagnosis.

*Liphistiusbuyphradi* sp. nov. is similar to *L.kaengkhoi* sp. nov., *L.champakpheaw* sp. nov., *L.hintung* sp. nov. and *L.suwat* in its general appearance. The male of *L.buyphradi* sp. nov. can be distinguished from those species, except for *L.champakpheaw* sp. nov., by the male palp with flat paracymbium (Fig. 14; Schwendinger 1996: fig. 43A); and from *L.champakpheaw* sp. nov. by the male palp with proximal edge of the tegulum moderately long, arched, and finely dentate (short, oblique in *L.champakpheaw* sp. nov.; Fig. 13C). The female *L.buyphradi* sp. nov. is difficult to distinguish from those of the *L.kaengkhoi* sp. nov., *L.champakpheaw* sp. nov., *L.hintung* sp. nov. and *L.suwat* Schwendinger, 1996. Molecular phylogeny and species delimitation using *COI* and multi-locus data support monophyly and species status of all new species described here (Sivayyapram et al. 2023).

##### Description.

**Male** (Holotype: ARA-2017-139; Fig. 10A, B). ***Coloration (in alcohol)***: carapace brown with black stripe along the margins; abdominal tergite olive green, except on the white posterior margins; membranous parts of the opisthosoma cream colored with black mottled spots; chelicerae olive green, paler on the proximal part; palp and legs olive green, without distinct annulation.

***Palp*** (Fig. 11A–D): tibial apophysis round, carrying one long slender and three tapering megaspines; paracymbium round but flat, dark patch with spicules isolated by a pale band; cumulus plane, bearing long black bristles; subtegulum without apophysis; contrategulum without apophysis, distal edge of contrategulum narrow with a depression leading to the oblique conical dorsal apex; tegulum large, axe-blade shaped, possessing a long ridge on the surface, indistinctly separated from the contrategulum, proximal margin with arched, moderately dentate edge, distal margin oblique with moderately apophysis; pigmental bridge between tegulum and contrategulum distinct; paraembolic plate round, as long as wide, project into scale like-plate; embolus proper: sclerotized part with two longitudinal ridges reaching to the tip.

***Measurements***: Total length 15.60; carapace 7.80 long, 7.28 wide; opisthosoma 8.19 long, 7.02 wide; ocular tubercle 1.04 long, 1.04 wide; palpal coxa 2.10 long, 1.17 wide; labium 0.72 long, 1.44 wide; sternum 3.92 long, 2.16 wide (1.02 on ventral surface); palp 12.48 long (4.14 + 2.34 + 3.90 + – + 2.10); leg I 20.88 long (6.06 + 3.00 + 4.26 + 5.22 + 2.34); leg II 22.62 long (6.24 + 3.12 + 5.16 + 7.14 + 2.64); leg III 24.66 long (6.60 + 3.12 + 5.16 + 7.14 + 2.64); leg IV 31.44 long (8.22 + 3.30 + 6.54 + 9.66 + 3.72).

**Female** (Allotype: ARA-2017-140; Fig. 10C, D). ***Coloration (in alcohol)***: carapace orange with black marking behind the ocular tubercle and on the peripheral area of the thoracic region; abdominal tergites black, except on the white posterior margins; membranous part of the opisthosoma cream colored with black mottled spots; chelicerae orange, paler at the proximal part; palp and leg femora to metatarsi orange, distal part of metatarsi I–III with black mottled marking, tarsi I–III black, tarsi IV orange with black annulations on the proximal and distal parts.

**Figure 12.**
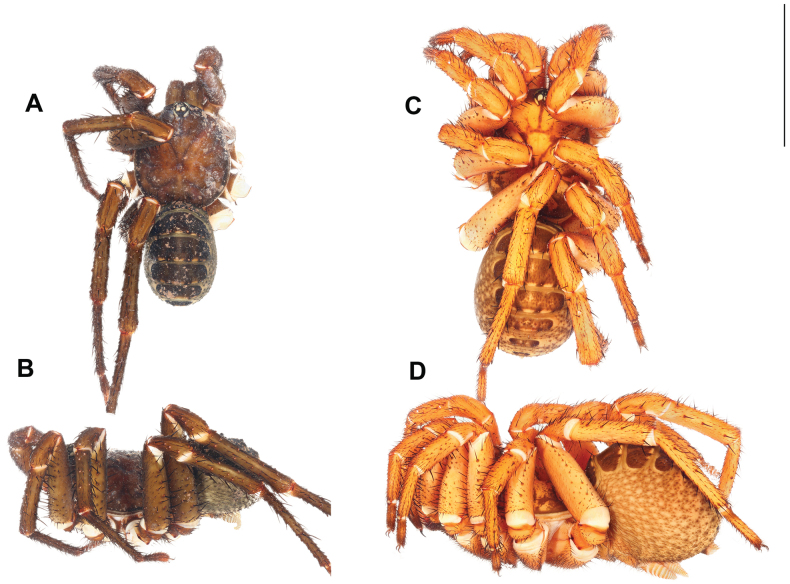
*Liphistiuschampakpheaw* sp. nov. **A, B** male ARA-2017-146 (holotype) **C, D** female, ARA-2017-146a (allotype) **A, C** dorsal view **B, D** lateral view. Scale bar: 10 mm.

***Vulva*** (Fig. 12E, F): vulva plate almost round; genital atrium with lateral hairs; posterior stalk narrow, V-shaped; pore plate quadrangular slightly wider than long; lateral margins thickened, projected into a lip, bearing moderate anterolateral lobes; anterior margin arched, thickened, not projected into a lip; receptacular cluster racemose; central dorsal opening wide.

***Measurements***: Total length 24.70; carapace 10.66 long, 8.97 wide; opisthosoma 12.35 long, 8.84 wide; ocular tubercle 1.04 long, 1.04 wide; palpal coxa 3.24 long, 2.88 wide; labium 1.16 long, 2.48 wide; sternum 5.36 long, 2.80 wide (1.76 on ventral surface); palp 16.90 long (5.80 + 3.40 + 3.90 + – + 3.80); leg I 20.20 long (6.90 + 3.90 + 4.00 + 3.50 + 1.90); leg II 20.40 long (6.60 + 3.80 + 4.10 + 4.10 + 1.80); leg III 22.30 long (6.80 + 4.00 + 4.10 + 5.00 + 2.40); leg IV 30.80 long (8.40 + 4.20 + 6.10 + 8.20 + 3.90).

##### Etymology.

The specific epithet *buyphradi* is dedicated to Mr. Phuri Buyphrad for providing information on the type locality of the new species.

##### Distribution.

Known only from the type locality.

##### Comment.

This new species name was mentioned as *Liphistius* sp. SL in Sivayyapram et al. (2023).

#### ﻿ Liphistius champakpheaw

Taxon classificationAnimaliaAraneaeLiphistiidae

Sivayyapram & Warrit sp. nov.

EF63018D-2171-5F27-81E2-5C40AF10B29D

https://zoobank.org/AB4110C0-8320-4090-BDAA-8C390A0584D4

**Figure 13.**
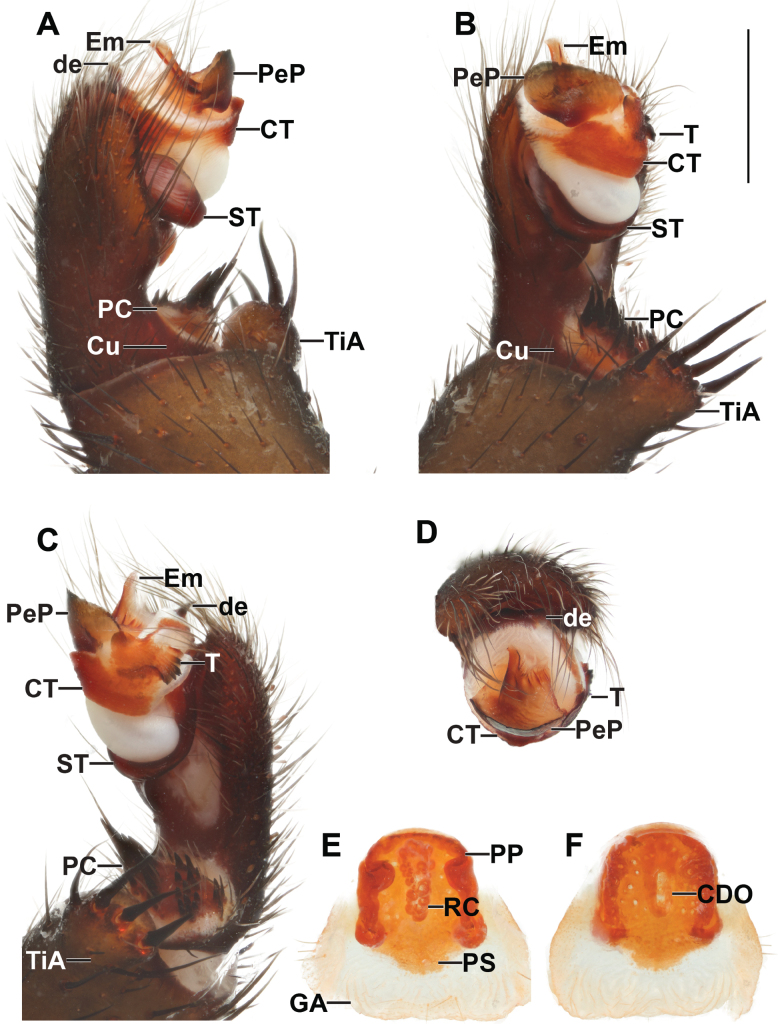
*Liphistiuschampakpheaw* sp. nov. male palp and vulva plate **A–D** ARA-2017-146 (holotype) palp **A** prolateral view **B** ventral view **C** retrolateral view **D** distal view **E, F** ARA-2018-146a (allotype) vulva plate **E** ventral view **F** dorsal view. Abbreviations: CDO = central dorsal opening; CT = contrategulum; Cu = cumulus; de = distal edge of the contrategulum; Em = embolus; GA = genital atrium; mm = millimeter; PC = paracymbium; PeP = paraembolic plate; PP = poreplate; PS = posterior stalk; RC = receptacular cluster; ST = subtegulum; T = tegulum; TiA = tibial apophysis. Scale bar: 1 mm.

**Figure 14.**
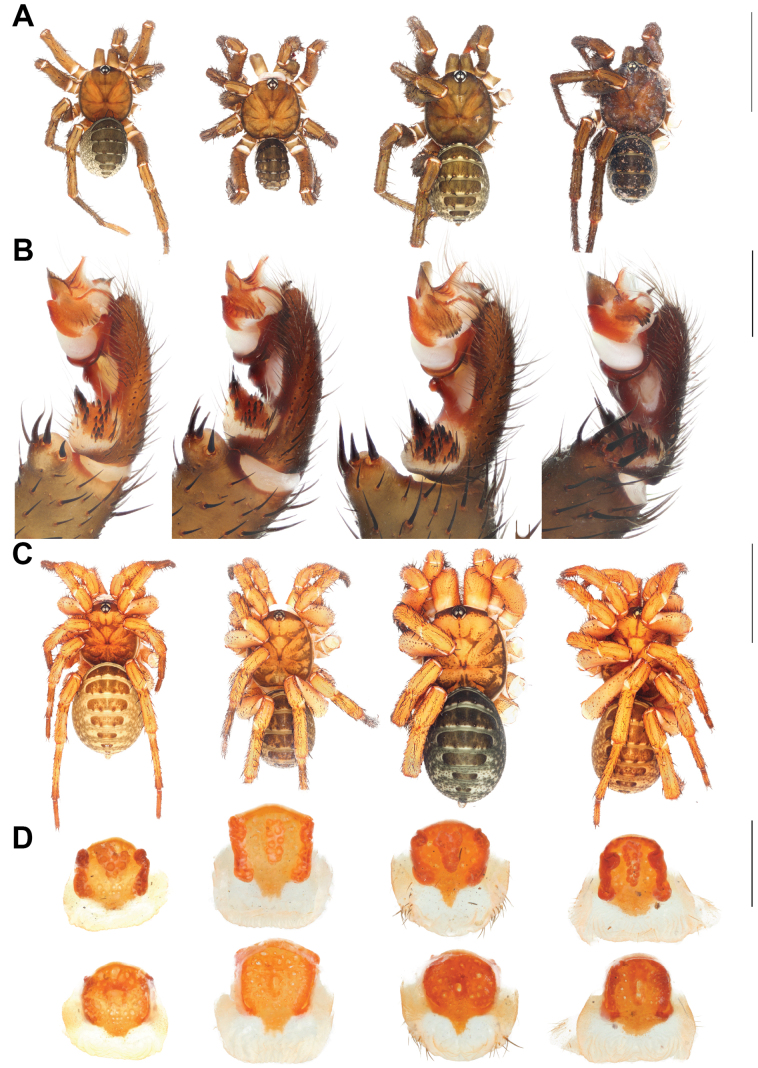
Left to right: *Liphistiuskaengkhoi* sp. nov., *Liphistiushintung* sp. nov., *Liphistiusbuyphradi* sp. nov., and *Liphistiuschampakpheaw* sp. nov. **A** male dorsal view **B** male palp **C** female dorsal view **D** vulva plate. Scale bars: 10 mm (**A, C**); 1 mm (**B, D**).

Figs 1 , 12 , 13 , 14

##### Type material.

***Holotype***: Thailand • 1♂; Saraburi, Kaeng Khoi District, Cham Phak Phaeo; alt. 82 m; 19 August 2017; N. Warrit, V. Sivayyapram, C. Kunsete, N. Chatthanabun, P. Traiyasut leg.; ARA-2017-146. ***Allotype***: Thailand • 1♀; same data as for the holotype; ARA-2017-146a.

##### Diagnosis.

*Liphistiuschampakpheaw* sp. nov. is similar to *L.kaengkhoi* sp. nov., *L.hintung* sp. nov., *L.buyphradi* sp. nov., and *L.suwat* in its general appearance. *Liphistiuschampakpheaw* sp. nov. is closely similar to *L.buyphradi* sp. nov. but can be distinguished by the male palp with tegulum that is short, obliqued, with dentate proximal edge (moderately long, arched in *L.buyphradi* sp. nov., Fig. 11C). The female *L.champakpheaw* sp. nov. is difficult to distinguish from those of the *L.kaengkhoi* sp. nov., *L.hintung* sp. nov., *L.buyphradi* sp. nov., and *L.suwat* Schwendinger, 1996. Molecular phylogeny and species delimitation using *COI* and multi-locus data support monophyly and species status of all new species described here (Sivayyapram et al. 2023).

##### Description.

**Male** (Holotype: ARA-2017-146; Fig. 12A, B). ***Coloration (in alcohol)***: carapace brown, slightly paler in the central area, without any distinct markings; abdominal tergites black, except for cream colored spots on the posterior margin; membranous part of the opisthosoma cream colored with black mottled spots; chelicerae olive green, paler at the proximal part; palp and legs uniformly brown.

***Palp*** (Fig. 13A–D): tibial apophysis wide distally, carrying four tapering megaspines; paracymbium round but flat, dark patch with spicules isolated by a pale band; cumulus plain, bearing several long bristles; subtegulum without apophysis; contrategulum without apophysis, distal edge of the contrategulum long with slight concavity leading to the truncate apex; tegulum moderate, proximal margins with short, tooth-like coarsely dentate edge; distal margin oblique, with large apophysis; paraembolic plate projected into scale-like plate, large, basally wide, running to blunt distal margin; embolus proper: sclerotized part with two longitudinal ridges reaching to the tip.

***Measurements***: Total length 14.82; carapace 7.54 long, 7.02 wide; opisthosoma 7.41 long, 5.46 wide; ocular tubercle 1.17 long, 1.43 wide; palpal coxa 2.10 long, 1.17 wide; labium 0.78 long, 1.30 wide; sternum 3.90 long, 3.64 wide (1.17 on ventral surface); palp 12.22 long (4.03 + 1.56 + 4.42 + – + 2.21); leg I 23.53 long (6.89 + 2.86 + 5.07 + 6.24 + 2.47); leg II 24.83 long (7.02 + 2.99 + 5.07 + 6.76 + 2.99); leg III 28.34 long (7.28 + 3.38 + 5.72 + 8.32 + 3.64); leg IV 34.84 long (8.84 + 3.51 + 7.15 + 10.92 + 4.42).

**Female** (Allotype: ARA-2017-146a; Fig. 12C, D). ***Coloration (in alcohol)***: carapace orange with black markings on the coxal elevations and the carapace margins; abdominal tergites with black markings except for the cream color on the posterior margin; membranous part of the opisthosoma cream colored with mottled black spots; chelicerae orange; palp and legs orange with black annulation on the proximal and distal part of tarsi.

***Vulva*** (Fig. 13E, F): vulva plate triangular, as long as wide; genital atrium with a few hairs and wrinkle posterior margin; posterior stalk U-shape, short but wide; pore plate quadrangular, slightly longer than wide; lateral margins thickened and projected into lips, bearing moderate anterolateral lobe; anterior margin arched, less thickened but not projected into a lip; receptacular cluster racemose longer than wide; central dorsal opening longer than wide.

***Measurements***: Total length 19.37; carapace 9.23 long, 7.41 wide; opisthosoma 9.88 long, 8.06 wide; ocular tubercle 1.17 long, 1.56 wide; palpal coxa 3.25 long, 2.08 wide; labium 1.17 long, 2.47 wide; sternum 5.07 long, 2.86 wide (1.69 on ventral surface); palp 15.73 long (5.59 + 3.12 + 3.77 + – + 3.25); leg I 18.72 long (6.24 + 3.51 + 3.77 + 3.38 + 1.82) leg II 18.59 long (5.72 + 3.38 + 3.77 + 3.90 + 1.82); leg III 20.15 long (6.11 + 3.38 + 3.90 + 4.42 + 2.34); leg IV 28.99 long (8.32 + 3.90 + 5.59 + 7.80 + 3.38).

##### Etymology.

The specific epithet *champakpheaw* refers to Cham Pak Pheaw subdistrict, the type locality of the new species in Saraburi, Thailand.

##### Distribution.

Known only from the type locality.

##### Comment.

This new species name was mentioned as *Liphistius* sp. CPP in Sivayyapram et al. (2023).

## ﻿Discussion

Here we described seven new *Liphistius* species can be assigned to two species groups, the *bristowei* group and the *trang* group, based on the characteristics of male and female genitalia.

### ﻿The *bristowei* group

*Liphistiusdawei* sp. nov. is assigned to the *bristowei* group by the male palp with an elevated cumulus, adjoining the embolus with the sclerotized part bearing two longitudinal ridges reaching to the tip, the paraembolic plate not projected into a scale-like plate, the subtegulum with an apophysis; and the vulva with projected corners of the pore plate. However, the vulva bears a unique posterior stalk constricted at the base which is more similar to the species belonging to the *birmanicus* group. *Liphistiuschoosaki* sp. nov. and *L.lansak* sp. nov. are also assigned to the *bristowei* group according to the vulva plate having a wide posterior stalk. The males of the two latter species are unknown.

### ﻿The *trang* group

*Liphistiuskaengkhoi* sp. nov., *L.hintung* sp. nov., *L.buyphradi* sp. nov., and *L.champakpheaw* sp. nov. are assigned to the *trang* group based on the characters of a detached embolus, a paraembolic plate projected to scale-like plate, the tegulum with a prominent distal margin, the subtegulum without an apophysis; and the vulva with small central dorsal opening and receptacular clusters. Specifically, all species are attributed to the complex A of the *trang* group according to the male palp with a plain cumulus, the contrategulum without an apophysis; and the female with an orange carapace and femora, the vulva with a square pore plate, a U-shape posterior stalk, and a racemose receptacular cluster.

## Supplementary Material

XML Treatment for Liphistius

XML Treatment for Liphistius dawei

XML Treatment for Liphistius choosaki

XML Treatment for Liphistius lansak

XML Treatment for Liphistius kaengkhoi

XML Treatment for Liphistius hintung

XML Treatment for Liphistius buyphradi

XML Treatment for Liphistius champakpheaw

## ﻿Additional information

### Author ORCIDs

Varat Sivayyapram https://orcid.org/0000-0002-8466-0270

Xin Xu https://orcid.org/0000-0001-5632-6622

Deborah R. Smith https://orcid.org/0000-0002-2581-5009

Hirotsugu Ono https://orcid.org/0000-0002-8568-4549

Daiqin Li https://orcid.org/0000-0001-8269-7734

Natapot Warrit https://orcid.org/0000-0002-6338-1782

### Data availability

All of the data that support the findings of this study are available in the main text.
